# Multisensory training can promote or impede visual perceptual learning of speech stimuli: visual-tactile vs. visual-auditory training

**DOI:** 10.3389/fnhum.2014.00829

**Published:** 2014-10-31

**Authors:** Silvio P. Eberhardt, Edward T. Auer Jr., Lynne E. Bernstein

**Affiliations:** Communication Neuroscience Laboratory, Department of Speech and Hearing Sciences, George Washington UniversityWashington, DC, USA

**Keywords:** multisensory perception, speech perception, reverse hierarchy theory, lipreading, vibrotactile perception, vocoded speech, perceptual learning

## Abstract

In a series of studies we have been investigating how multisensory training affects unisensory perceptual learning with speech stimuli. Previously, we reported that audiovisual (AV) training with speech stimuli can promote auditory-only (AO) perceptual learning in normal-hearing adults but can impede learning in congenitally deaf adults with late-acquired cochlear implants. Here, impeder and promoter effects were sought in normal-hearing adults who participated in lipreading training. In Experiment 1, visual-only (VO) training on paired associations between CVCVC nonsense word videos and nonsense pictures demonstrated that VO words could be learned to a high level of accuracy even by poor lipreaders. In Experiment 2, visual-auditory (VA) training in the same paradigm but with the addition of synchronous vocoded acoustic speech impeded VO learning of the stimuli in the paired-associates paradigm. In Experiment 3, the vocoded AO stimuli were shown to be less informative than the VO speech. Experiment 4 combined vibrotactile speech stimuli with the visual stimuli during training. Vibrotactile stimuli were shown to promote visual perceptual learning. In Experiment 5, no-training controls were used to show that training with visual speech carried over to consonant identification of untrained CVCVC stimuli but not to lipreading words in sentences. Across this and previous studies, multisensory training effects depended on the functional relationship between pathways engaged during training. Two principles are proposed to account for stimulus effects: (1) *Stimuli presented to the trainee’s primary perceptual pathway will impede learning by a lower-rank pathway*. (2) *Stimuli presented to the trainee’s lower rank perceptual pathway will promote learning by a higher-rank pathway*. The mechanisms supporting these principles are discussed in light of multisensory reverse hierarchy theory (RHT).

## Introduction

Several studies have demonstrated that audiovisual (AV) training can promote perceptual learning of degraded auditory-only (AO) speech beyond training with AO stimuli (Pilling and Thomas, 2011; Wayne and Johnsrude, 2012; Bernstein et al., 2013, 2014; Huyse et al., 2013). Audiovisual training has also been shown to promote learning a phonemic contrast in a second language (Hardison, 2003; Hazan et al., 2006). Visual speech information could be beneficial to auditory perceptual learning, because concordant or correlated visual speech information (Yehia et al., 1998; Jiang et al., 2002) could guide the learning of new auditory speech representations (Rouger et al., 2007).

More generally, AV speech promoting auditory perceptual learning could be one case of a class of perceptual learning contexts in which concordant or correlated stimuli to one sensory system assists perceptual learning by a different system. Another example is the use of an intact sensory system to guide learning with a sensory prosthesis for a disordered system. For example, sensory-guided plasticity using auditory or vibrotactile perception has been suggested as a possible approach to enhancing perceptual learning with a visual prosthesis (Merabet et al., 2005; Proulx et al., 2014). This suggestion is consistent with findings from psychophysical training experiments that show better visual perception after training with concordant acoustic patterns (Shams et al., 2011; van Wassenhove, 2013; Zilber et al., 2014). Such results encourage the view that multisensory stimuli are consistently useful in promoting unisensory perceptual learning.

However, a recent study (Bernstein et al., 2014) with prelingually deafened adults who obtained auditory prostheses—cochlear implants—late into development and mostly as adults showed that AV training actually impeded perceptual learning of AO speech stimuli. Participants had the task to learn lists of 12 pairs of associations between disyllabic (C = [consonant] V = [vowel]CVC) nonsense words and nonsense pictures (*fribbles*) (Williams and Simons, 2000). They were assigned to train with AV or AO stimuli in two different orders and were always tested with AO stimuli. The results showed that whenever the training stimuli were AV, the AO test scores were dramatically lower. Paired-associates training with AO stimuli resulted in similar or somewhat higher AO test scores. These results contrasted with ones from a group of normal-hearing adults who carried out the same experiment, except that instead of training on speech presented via a cochlear implant, they trained with vocoded acoustic speech. Their auditory perceptual learning was not impeded and was even promoted to an extent, consistent with earlier results (Bernstein et al., 2013). Thus, there is evidence that multisensory stimuli are not always useful and indeed can impede learning.

One possible explanation for these findings is that the prelingually deafened cochlear implant users learned differently due to neuroplastic changes associated with deafness (Kral and Eggermont, 2007; Kral and Sharma, 2012). Indeed, lipreading is significantly better in deaf adults and children who rely on spoken language than in normal-hearing individuals (Bernstein et al., 2000; Mohammed et al., 2005; Auer and Bernstein, 2007; Tye-Murray et al., 2014). Lipreading ability is also highly stable in both deaf and hearing adults (Bernstein et al., 2001). The impeder effect of multisensory training observed with prelingually deafened late-implanted cochlear implant users could be a consequence of enhanced visual speech perception ability in the context of developmental auditory deficits.

However, in our study we also obtained evidence that the cochlear implant users allocated their attention differently than did normal-hearing adults performing the same training protocol. The cochlear implant users were most accurate in identifying initial consonants in untrained CVCVC auditory stimuli, while the normal-hearing adults were most accurate for medial consonants. The cochlear implant users appeared to use a lipreading strategy seen in consonant identification by deaf and hearing lipreaders (Auer and Bernstein, in preparation). Normal-hearing listeners appeared to be biased towards medial consonants, likely because additional acoustic phonetic information is available at intervocalic positions by the vowel transitions into and out of the consonant (Stevens, 1998). Interestingly, the cochlear implant users at pre-training were even more accurate than normal-hearing adults at identifying initial consonants, suggesting that had they known how to attend to the intervocalic consonant stimuli, they could have benefited more from training. Thus, the cochlear implant group could have been impeded by neuroplastic changes, general perceptual biases toward visual stimuli, and even biases that controlled their attention within the fine structure of the stimuli.

On the other hand, the results from the cochlear implant users led us to consider whether multisensory training should be symmetric, that is, should it be beneficial to an extent to both modal systems (i.e., visual and auditory) in neurotypical individuals? If exposure to AV speech had symmetric effects on visual speech perception, we might expect there to be little difference in lipreading between normal-hearing and deaf adults, as normal-hearing individuals are constantly being presented with visual speech. Theories about statistical learning suggest that the prevalent environmentally available speech patterns are learned (Saffran et al., 1996; Abla and Okanoya, 2009; Shams and Kim, 2010). If AV exposure can benefit AO perception, why are normal-hearing lipreaders generally poor? They are constantly being exposed to visual speech patterns, and the excellent lipreaders among deaf individuals suggest that those patterns are learnable.

In this study, we therefore sought to demonstrate that multisensory training can promote or impede unisensory visual speech perceptual learning in normal-hearing adults, and that the promoter or impeder effects are related to what we refer to as the “rank” of the perceptual system receiving the stimuli. Normal-hearing individuals rely on auditory speech perception as their primary modality and have—to varying individual extent—ability to perceive visual speech stimuli, which are received via their secondary rank modality for speech. They are not expected to have experienced vibrotactile vocoded speech, but they have experienced somatomotor feedback from their own speech production, including stimulation from laryngeal vibration, the breath stream, and kinesthesia. Thus, vibrotactile speech stimuli are considered to be of tertiary rank. The stimuli examined here as potential promoters or impeders of visual speech perceptual learning were vocoded auditory and vibrotactile speech, respectively.

### Lipreading

Throughout the twentieth century, studies were carried out on lipreading ability and training in deaf children and adults (Nitchie, 1912; Heider and Heider, 1940; Utley, 1946; Jeffers and Barley, 1971; Conrad, 1977). Before the advent in the 1980s of cochlear implants, which stimulate the auditory nerve directly (Zeng et al., 2004), development of good lipreading skills was a critical goal for deaf children. However, training could not be relied upon to confer accurate lipreading, leading to the view that good lipreaders are born and not made (Heider and Heider, 1940). Another view was that normal hearing is required to achieve the highest lipreading levels possible, because according to the argument, lipreading relies on having a language system established via the auditory system (Mogford, 1987; Rönnberg et al., 1998). Overall, lipreading training has been regarded as not very effective (Massaro, 1987; Mogford, 1987; Summerfield, 1991).

Nevertheless, modest improvements in various lipreading tasks have been reported following training (Walden et al., 1977, 1981; Gesi et al., 1992; Massaro et al., 1993). In Gesi et al. (1992), normal-hearing college students trained on consonant-vowel (CV) nonsense syllables and improved their identification scores, but training did not transfer to identification of monosyllabic words. Massaro et al. (1993) followed up with a training study that used the same tokens of CV syllables, monosyllabic words, and sentences presented audiovisually, visual-only (VO), and AO. Although there were improvements in VO perception, the use of the same stimuli across all conditions precludes attributing improvements to perceptual learning of visual speech phonemes or features as opposed to memory for the specific items. Studies by Walden et al. (1981) used training on VO consonants and showed improved consonant perception (10%), including improved (23%) AV perception of sentences in adults with hearing impairments. Sentence lipreading was not studied.

There is evidence in the literature for relatively large improvements (10–30 percentage points) in lipreading in the context of training with vibrotactile speech stimuli (DeFilippo and Scott, 1978; DeFilippo, 1984; Weisenberger et al., 1989; Eberhardt et al., 1990; Bernstein et al., 1991; Waldstein and Boothroyd, 1995; Kishon-Rabin et al., 1996). The findings come from research on vibrotactile speech devices that were invented to supplement lipreading by deaf people but were mostly tested with normal-hearing adults.

Vibrotactile devices comprise an input signal transducer (microphone or line in), signal processing, and an output with one or more small vibrators that synchronously present some attributes of the acoustic input (Summers, 1992). Lipreading scores were used as the baseline measure, and scores from tests with vibrotactile and visual stimuli combined were used to test device effects. Although intended to supplement lipreading, the combined lipreading and vibrotactile device training (with normal-hearing participants) sometimes led to lipreading learning that actually impeded demonstrating device effectiveness, because the lipreading improvements were as large, or larger, than the effects of the devices. Visual-tactile (VT) performance improved, but the effect generally rode on top of an increasing VO baseline.

The VO gains were fairly impressive. In Eberhardt et al. (1990), the VO gain in lipreading words in sentences was 16 percentage points; in another study (Kishon-Rabin et al., 1996) the VO improvement was as high as roughly 30 percentage points; and similarly in yet another study (Bernstein et al., 1991), pre- to post-training scores exhibited an average 24 percentage point gain in lipreading (see also Weisenberger et al., 1989; Waldstein and Boothroyd, 1995). However, such magnitudes of gain were not seen universally. In Bernstein et al. (2001), hearing and deaf adults lipread sentences with feedback over several sessions. Some of the participants also received vibrotactile speech stimuli during sentence lipreading, although there was no explicit training for vibrotactile-only stimuli. There were small but reliable declines in VO sentence lipreading when participants had received VT training. A possible explanation was that trainees integrated the visual and vibrotactile stimuli and paid less attention to the visual modality whenever the VT stimuli were presented.

Overall, results on lipreading training in adults with normal auditory development suggest that some gains can be achieved, that the gains can generalize to materials outside of training, and that vibrotactile stimuli can promote learning that exceeds training with VO stimuli, although we have obtained counter-evidence as well.

### The current study

A series of visual speech training experiments was designed to investigate whether perceptual learning with multisensory (visual-auditory or visual-tactile^1^) vs. unisensory VO stimuli follows general principles that apply across different combinations of multisensory speech stimuli for unisensory perceptual learning. Our hypothesis was that unisensory (here, visual) speech perceptual learning can be promoted when the training conditions provide a concurrent stimulus that is delivered via a modality that has a lower perceptual rank (here, vibrotactile) for the speech perception task; and that unisensory perceptual learning for speech can be impeded under multisensory training conditions when the training conditions provide a concurrent stimulus that is delivered via the trainee’s primary speech perception modality (here, auditory). A no-training control experiment was carried out to help interpret pre- and post-training scores on identification of consonants in untrained CVCVC stimuli and lipreading of words in sentences. The outcomes of the four training experiments, which are consistent with our hypothesis about promoter vs. impeder stimuli, have implications for clinical and other practical speech training applications. The results are discussed in the context of a theoretical account of how multisensory mechanisms are engaged in terms of reverse hierarchy theory (RHT; Hochstein and Ahissar, 2002; Ahissar et al., 2009).

## General methods

This section describes methods that were applied across experiments. Methods specific to only one particular experiment are described with that experiment.

### Participants

Participants were screened for lipreading ability using a sentence lipreading task that we routinely use for this purpose (Auer and Bernstein, 2007). Seventy-nine participants were recruited, and three dropped out citing lack of time. The participants were assigned to the five experiments, so that mean lipreading scores were similar across groups. This approach was deemed necessary in light of the wide individual differences among lipreaders (Bernstein et al., 2000; Auer and Bernstein, 2007). The participants were assigned to different groups across experiments. Groups were: Experiment 1, VO (*N* = 20, ages 18–31 years, mean 21.9 years, 2 male); Experiment 2, visual-auditory (VA) (*N* = 13, ages 19–22 years, mean 20.7 years, 4 male); Experiment 3, AO (*N* = 8, ages 19–26 years, mean 22.2 years, 5 male); Experiment 4, VT (*N* = 21, ages 19–34 years, mean 23.0 years, 2 male); and Experiment 5, no-training controls (*N* = 13, ages 19–27 years, mean 22.2 years, 2 male). All participants signed an informed consent form that was approved by the George Washington Institutional Review Board. They were all paid for their participation.

### CVCVC nonsense word stimuli

The audio and video source recordings used were described previously (Bernstein et al., 2013, 2014). The following is an abbreviated description.

The spoken CVCVC nonsense words used for the paired-associates training and testing paradigm, as well as for the pre- and post-training consonant identification task with untrained stimuli, were modeled on English phonotactics (i.e., the sequential speech patterns in English) using Monte Carlo methods. There were 260 unique words, which were recorded from a female talker. All of the words were theoretically visually distinct for lipreading and also visually unique from real English words (i.e., the words were designed to not be mistaken as real words, if they were lipread without accompanying audio). For example, the nonsense word *mucker* was not included in the set, because the visual stimulus could be mistaken for the real word *pucker*, inasmuch as the phonemes /p, m/ are visually highly similar (Auer and Bernstein, 1997). The visual distinctiveness of the words was shown empirically for the first time in the present study. The full set of nonsense words includes all the English phonemes.

Four lists of 12 CVCVC words for paired-associates training and four lists of six words as new items during paired-associates testing were selected from the available words as were two 49-item lists pre- and post-training consonant identification. Two six-item lists were selected for initial practice with pre- and post-training CVCVC consonant identification and sentence lipreading. Word lists were the Training and Test Lists 1–4 from Table 3 in Bernstein et al. (2013).

### Nonsense pictures

The nonsense pictures in the paired-associates paradigm were from the fribbles image set^2^ and were used in previous experiments (Bernstein et al., 2013, 2014). Fribbles comprise 12 species with distinct body “core” shape and color, with 81 exemplars per specie obtained by varying the forms of each of four appendage parts. From the available images, four lists of 12 images each were created such that each list used three different body forms and no duplicated appendage forms, rendering the images within each list highly distinctive (Williams and Simons, 2000). No appendage was repeated across lists.

### Isolated sentences

Two different 50-sentence lists comprised video recordings of IEEE sentences (IEEE, 1969). The lists were compiled based on a pilot lipreading experiment that was used to generate lists with equal expected mean scores. Participants received the lists in counterbalanced order within experiments.

### Overall design of the procedures

**Figure 1:**
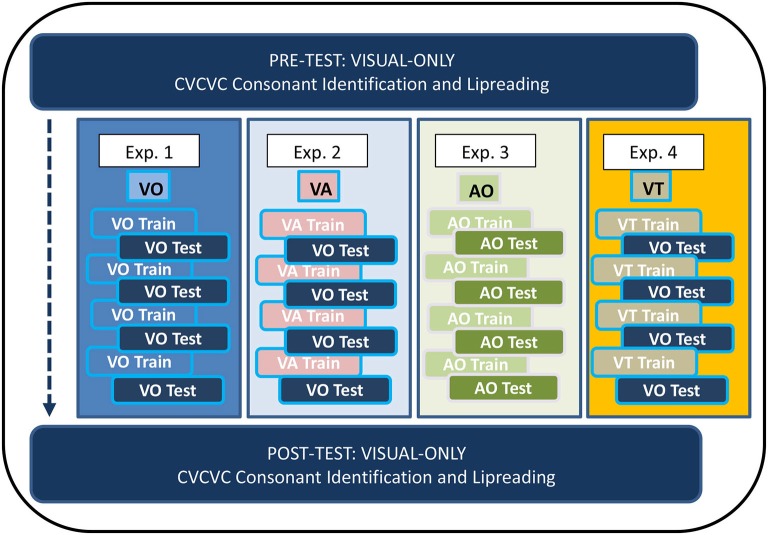
**Paired-associates training procedure**. The four lists of paired-associations in this study were the same across all training (Experiments 1–4). There was no counter-balancing for list order. All participants received visual-only pre-training and post-training tests for consonant identification with untrained CVCVC stimuli and for lipreading words in sentences.

Figure 1 shows the overall design of the experiments in which participants received training. In Experiments 1–4, participants trained on four lists of 12 paired associations (each list on a different day) with training stimuli that were VO (Exp. 1), VA (Exp. 2), AO (Exp. 3), or VT (Exp. 4). Paired-associates testing (on the same day as training) was either VO (Exps. 1, 2, and 4) or AO (Exp. 3). Participants carried out consonant identification with untrained CVCVC nonsense words on two occasions corresponding to pre-training and post-training, and they also identified words in sets of 50 unrelated sentences on the same occasions. The no-training control subjects in Experiment 5 carried out on different days only the pre and post-training tasks.

**Figure 2:**
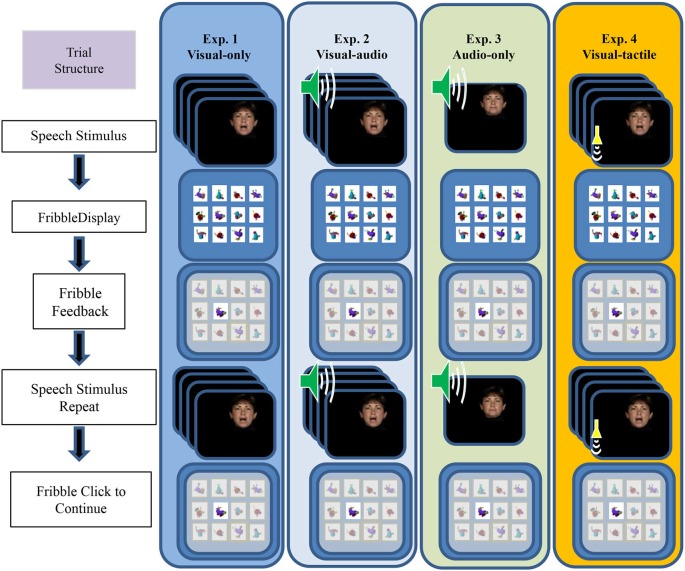
**Sequence of events during a paired-associates training trial**. The speech stimulus was presented, followed by the matrix of fribble responses, followed by the participant’s response selection, followed by feedback and a repetition of the speech stimulus. The next trial was initiated by clicking on the correct highlighted fribble. Panels depict the stimuli for the four experiments from left to right. The trial structure for VO (Exp. 1), VA (Exp. 2), AO (Exp. 3), and VT (Exp. 4) training followed the same sequence, except that during AO training with vocoded audio the participant was not shown a moving face. During VA the visual stimuli were accompanied by vocoded audio, and during VT the visual stimuli were accompanied by vibrotactile vocoder stimuli (adapted from Bernstein et al., 2013).

Figure 2 outlines the events within a paired-associates training trial. During training, the participant’s task was to learn, by trial and error with feedback on each trial, lists of individual associations between each of 12 CVCVC spoken nonsense words and 12 fribble images. The figure shows the four different types of training conditions, VO (Exp. 1), VA (Exp. 2), AO (Exp. 3), and VT (Exp. 4). Figure 2 shows that each trial was initiated by presenting a speech stimulus then the 12-fribble image matrix (3 rows of 4 columns, with image position within the matrix randomly selected on a trial-by-trial basis). The participant selected a fribble image and the screen darkened except for the correct response. The participant received the stimulus again, and after the stimulus was presented again, the participant clicked on the correct fribble in order to move on to the next trial.

A training block comprised two repetitions of the 12 paired associations in pseudorandom order. There were three blocks per training list. Thus, training for each association was given on six trials. The training score was the proportion of correctly paired associations of trained words in the block. Prior to the first training list, participants were given practice with one block of six trials.

### Paired-associate testing procedure

Paired-associates testing followed training after a few minutes’ rest period. The testing procedure was similar to that of training, except the CVCVC speech stimuli were always presented VO (except for Exp. 3), no feedback was given, the stimulus was not repeated during the trial, and each response triggered the next trial. Six of the trained words and all 12 of the fribble images were used for testing. The associations for the six retained words were unchanged. Six foil CVCVC nonsense words were paired with the fribble images of the discarded words. A testing block comprised, in pseudorandom order, four presentations of the 12 stimuli. The test score was the proportion of correctly paired associations of the six originally-trained words across all trials.

### CVCVC consonant identification

In a forced choice paradigm, participants identified the three consonants in 49 different untrained CVCVC stimuli before their first paired-associates training block (pre-) and after their fourth paired associates training test (post-). These CVCVC stimuli were all different from those in the paired-associates training paradigm.

The CVCVC stimuli had varied vowels that were not identified and 24 possible consonants transcribed using ARPABET single key-press transcriptions, /b, d, f, g, h, k, l, m, n, p, r, s, t, v, w, y, z, C, D, G, J, S, T, Z/ (which correspond to the International Phonetic Alphabet, /b, d, f, g, h, k, l, m, n, p, r, s, t, v, w, j, z, t*∫*, ð, η, dƷ, *∫*, Θ Ʒ/). In order to familiarize participants with the transcription set, they were given two computerized training sessions, one with an answer key that showed each of the ARPABET symbols, and one without the key. Each key item showed a word with a consonant underlined, and the corresponding ARPABET symbol. Each training item similarly displayed a word with an underlined consonant; participants typed the ARPABET symbol they thought represented the sound. Only during learning to use the transcription set, pressing an incorrect key elicited a message that the selection was wrong, and asked that they try again.

During testing, the key list of ARPABET symbols and word examples was always displayed. The three consonant positions were marked on the computer screen with “___-___-___” and the participants used the keyboard to fill in the blanks. They could backspace and correct mistakes. They were given a practice list prior to starting the first test list. The two lists of CVCVC stimuli were counterbalanced across participants. The task resulted in a proportion correct score for each consonant position in the CVCVC stimuli.

The responses were also scored in terms of phoneme equivalence classes (Auer and Bernstein, 1997) correct. The groupings for the classes (see Table 1) were generated by analyzing behavioral consonant confusion data collected with consonants produced by the same talker as the one who produced the CVCVC stimuli. Separate data sets were collected for consonants in the initial, medial, and final positions with vowels /i/, /u/, /a/ and /x/ (reduced vowel) (Auer and Bernstein, in preparation). Phoneme equivalence classes were defined using the procedures specified in (Auer and Bernstein, 1997; Bernstein, 2012). Perceptual confusion matrices were formed from stimulus–response identification data. These confusion matrices were then transformed into similarity matrices by computing a phi-square statistic on every pair of stimulus phonemes (Iverson et al., 1998). The resulting phoneme similarity matrices were then analyzed using hierarchical cluster analysis using an average-linkage-within groups method for the clustering (Aldenderfer and Blashfield, 1984). A standard level (comparable to the “viseme”) (e.g., Binnie et al., 1976; Walden et al., 1977; Massaro et al., 2012) was extracted from the cluster structure when the minimum within-the cluster response was set equal to or greater than 75 percent. Table 1 shows the actual within-cluster response percentages that were obtained when the stimuli were clustered.

**Table 1:** **Consonant equivalence classes used for scoring pre- and post-training CVCVC consonant identification response**.

Consonant position	Percent within-cluster confusions	Equivalence class groups
Initial	82.9	[l] [C J S d h g k n s z t y] [w r] [D T][f v] [b p m]
Medial	78.0	[C J S Z] [d h g k l n s z t y] [w] [D T] [b p m] [f v]
Final	77.3	[m][b p][D T][f v] [C J S Z] [d g k ln s z t G]

*Groupings were derived based on clustering of consonant confusions with CVCVC nonsense words. Consonant notation is ARPABET. Percent within-cluster confusions are listed for the lowest level clustering. (Initial = initial consonant; Medial = medial consonant; Final = final consonant)*.

### Analyses

All of the responses from paired-associates training and testing, and also from the consonant identification task were converted into proportions correct and then arcsine transformed, $y=\sin^{-1}\left(\sqrt{p}\right)$, where *p* is the proportion correct. The score range following the arcsine transformation is 0 to 90. Statistics are reported on the arcsine transformed data, but tables, means, and figures present untransformed data to facilitate interpretation. Analyses were carried out with SPSS (IBM Statistics SPSS 22). Unless explicitly noted, only effects that were reliable at least at the level of *p* < 0.05 are reported.

Lipreading was scored in terms of words correct in sentences, and scores were converted into proportion words correct. The wide variation in lipreading screening scores was evaluated in several ways in relationship to the results of each experiment. When there was evidence that lipreading ability as measured by screening scores was related to experimental measures, screening scores were used as covariates to adjust for individual differences. The continuum of scores is exceedingly unlikely to represent a linear scale. For example, individuals with scores close to 20% correct are likely more similar to ones with scores of 0% correct than they are to individuals with scores of 40% correct or greater.

## Experiment 1: lipreading training

Experiment 1 was carried out to evaluate the ability to learn paired associations between spoken visual nonsense words and nonsense pictures. Although the stimuli had been designed to be visually distinct, they had never been tested to determine how well they could be learned as VO stimuli in the paired-associates training paradigm. Pre- and post-training consonant identification and lipreading tests were administered to determine whether the training on paired associations generalized to untrained stimuli in tasks that were not used during training.

### Stimuli

The stimuli were the above-described VO CVCVC speech recordings presented during paired-associates training and testing and during pre- and post-training tests of consonant identification, and sentences presented for lipreading.

### Procedure

The procedure followed the one described above as outlined in Figure 1.

### Results

#### Lipreading screening scores

Lipreading screening scores ranged between 2.3% and 30.5% correct. Therefore, a concern was whether lipreading ability influenced training or test scores in the paired-associates task, the pre- and post-test phoneme identification task, or the pre- and post-training lipreading task. Lipreading screening scores were submitted to bivariate correlation analysis for scores from each of the final training blocks and test blocks of the paired association task for the four training lists (i.e., 4 training and 4 test scores), and the pre- and post-test consonant identification scores (initial, medial, and final). Only the pre- and post-training consonant identification scores were reliably correlated with lipreading screening scores (correlations ranged between *r* of 0.52 and 0.65, *p* <= 0.018).

#### Paired-associates training scores

**Figure 3:**
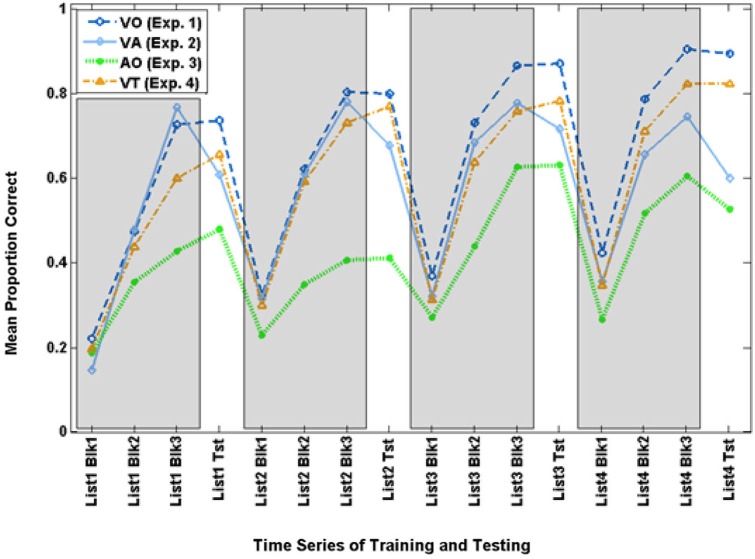
**Time series for mean paired-associates training and test scores across Experiments 1–4**. The three training block (Blk) scores for each list are in gray areas, and the test (Tst) scores are on the white background.

Figure 3 shows the time series for VO training and test scores, along with scores from Experiments 2–4.

The scores on the final VO block for each of the four training lists were submitted to a within-subjects analysis for list (4), *F*_(3,17)_ = 9.788, *p* = 0.001, $\eta_{p}^{2}$ = 0.633, which was shown to be reliable only for the linear trend, *F*_(1,19)_ = 28.054, *p* = 0.000, $\eta_{p}^{2}$ = 0.596, with scores increasing across lists from 72.5% correct to 90.3% correct.

#### Paired-associates test scores

The VO paired-associates test scores were submitted to a within-subjects analysis for list (4), which was not reliable in the multivariate analysis for list *F*_(3,17)_ = 2.387, *p* = 0.105, $\eta_{p}^{2}$ = 0.296, but was for the linear trend, *F*_(1,19)_ = 5.573, *p* = 0.029, $\eta_{p}^{2}$ = 0.227, with scores increasing across lists from 73.5% correct to 89.2%.

#### Pre- vs. post-training consonant identification

**Figure 4:**
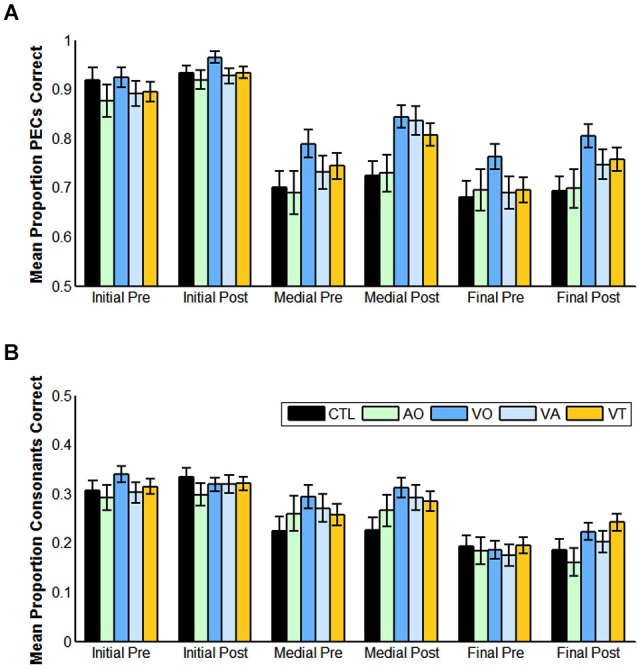
**Pre- and post-training consonant identification scores for each condition of training and for controls, and for each position (initial, medial, final) in the CVCVC stimuli**. These figures show the results averaged across all participants within each group. **(A)** Mean proportion phoneme equivalence classes (PECs) correct. **(B)** Mean proportion consonants correct. Note: Scales are different in **(A)** and **(B)** reflecting the more liberal scoring in **(A)**. Error bars are standard error of the mean.

Table 2 and Figure 4 gives pre- and post-training consonant identification mean scores for each of the consonant positions in terms of proportion consonants correct and proportion phoneme equivalence classes correct. The VO pre- and post-training consonant identification scores were submitted to a within-subjects analysis for position (initial, medial, or final in CVCVC stimuli) and test time (pre-, post-training). Lipreading screening scores were used as a covariate, because they correlated with the consonant identification scores. They were a reliable covariate, *F*_(1,18)_ = 11.529, *p* = 0.003, $\eta_{p}^{2}$ = 0.390. Position was a reliable factor, *F*_(2,17)_ = 39.832, *p* = 0.000, $\eta_{p}^{2}$ = 0.824, but so was its interaction with test time and the covariate, *F*_(2,17)_ = 5.152, *p* = 0.018, $\eta_{p}^{2}$ = 0.377. In simple comparisons, the interaction was isolated to the difference across time for the medial vs. final consonant positions *F*_(1,18)_ = 9.676, *p* = 0.006, $\eta_{p}^{2}$ = 0.350 (See Table 2 for all the consonant identification mean scores in each experiment, time period, and scoring approach).

**Table 2:** **Scores on pre- and post-training consonant identification with VO CVCVC stimuli**.

Group	Consonants correct scoring					
	Pre-training			Post-training		
	Initial	Medial	Final	Initial	Medial	Final
VO	0.340 (0.017)	0.294 (0.023)	0.186 (0.018)	0.319 (0.014)	0.313 (0.020)	0.223 (0.017)
VA	0.303 (0.021)	0.271 (0.028)	0.175 (0.022)	0.319 (0.018)	0.292 (0.025)	0.202 (0.022)
VT	0.315 (0.016)	0.257 (0.022)	0.195 (0.017)	0.321 (0.014)	0.285 (0.020)	0.242 (0.017)
AO	0.292 (0.026)	0.260 (0.036)	0.184 (0.028)	0.298 (0.023)	0.266 (0.032)	0.161 (0.028)
Control	0.306 (0.021)	0.225 (0.028)	0.194 (0.022)	0.335 (0.018)	0.227 (0.025)	0.185 (0.022)
Exp. 4 criterion-level sub-groups
VO	0.323 (0.021)	0.288 (0.029)	0.186 (0.018)	0.316 (0.015)	0.305 (0.021)	0.208 (0.021)
VT	0.328 (0.023)	0.287 (0.032)	0.185 (0.019)	0.328 (0.016)	0.296 (0.022)	0.258 (0.023)
Exp. 5
Visual training	0.321 (0.010)	0.274 (0.014)	0.187 (0.010)	0.320 (0.009)	0.297 (0.012)	0.225 (0.011)
No visual training	0.300 (0.016)	0.238 (0.022)	0.190 (0.017)	0.321 (0.014)	0.242 (0.020)	0.176 (0.017)
**Group**	**Phoneme equivalence class scoring**					
	**Pre-training**			**Post-training**		
	**Initial**	**Medial**	**Final**	**Initial**	**Medial**	**Final**
VO	0.924 (0.021)	0.789 (0.028)	0.763 (0.026)	0.965 (0.012)	0.844 (0.023)	0.805 (0.024)
VA	0.891 (0.026)	0.731 (0.034)	0.689 (0.033)	0.927 (0.015)	0.836 (0.029)	0.747 (0.030)
VT	0.895 (0.020)	0.744 (0.027)	0.695 (0.026)	0.934 (0.012)	0.807 (0.023)	0.758 (0.024)
AO	0.876 (0.033)	0.689 (0.044)	0.695 (0.042)	0.919 (0.019)	0.729 (0.037)	0.698 (0.039)
Control	0.919 (0.026)	0.700 (0.034)	0.681 (0.033)	0.933 (0.015)	0.725 (0.029)	0.693 (0.030)
Exp. 4 criterion-level sub-groups
VO	0.914 (0.033)	0.777 (0.032)	0.755 (0.028)	0.973 (0.009)	0.842 (0.020)	0.801 (0.029)
VT	0.892 (0.036)	0.770 (0.035)	0.713 (0.031)	0.951 (0.010)	0.860 (0.022)	0.777 (0.031)
Exp. 5
Visual training	0.905 (0.013)	0.758 (0.017)	0.719 (0.016)	0.944 (0.007)	0.828 (0.014)	0.773 (0.015)
No visual training	0.903 (0.020)	0.696 (0.027)	0.686 (0.026)	0.928 (0.012)	0.727 (0.023)	0.695 (0.024)

*The table shows mean scores with standard error of the mean in parentheses in terms of proportion consonants correct and in terms of proportion phoneme equivalence classes correct. The overall means are given for the five groups across experiments (VO, VA, VT, AO, control), the VO and VT participants who achieved criterion performance in Experiment 4, and for the groups separated between those who experienced visual stimuli during training and those who did not (VO, VT, VA vs. AO and control in Exp. 5)*.

When the same analysis was applied to phoneme equivalence class scores, only the main effects of position, *F*_(2,17)_ = 28.207, *p* = 0.000, $\eta_{p}^{2}$ = 0.768, time of test, *F*_(1,18)_ = 6.875, *p* = 0.017, $\eta_{p}^{2}$ = 0.276, and the lipreading screening covariate, *F*_(2,18)_ = 5.540, *p* = 0.030, $\eta_{p}^{2}$ = 0.235, were reliable. The pre-training mean score was 82.5% correct, and the post-training score was 87.2% correct. Initial consonants were more accurate than medial (initial = 94.5% correct; medial = 81.7% correct), *F*_(1,18)_ = 58.854, *p* = 0.000, $\eta_{p}^{2}$ = 0.766, but the difference between medial and final was not reliable (*p* = 0.155).

#### Pre- vs. post-training lipreading words in sentences scores

The pre- vs. post-training lipreading scores were submitted to analysis including the between subjects list factor and the lipreading screening test covariate. There were not any reliable effects other than the lipreading screening score covariate (*p* = 0.000).

### Discussion

In Experiment 1, participants whose lipreading scores ranged widely, including participants who demonstrated almost complete lack of lipreading ability and ones who were relatively proficient (2% to 30% words correct lipreading screening), were able to learn paired associations involving spoken disyllabic visual nonsense words and nonsense pictures. There was not any reliable correlation between lipreading screening scores and final training block scores or test scores from the paired-associates task. Across paired-associates training and test scores, performance levels reached approximately 90% correct. This result suggests that poor ability to identify spoken words through lipreading is not a good indication of ability to learn to identify spoken words through the visual modality, a finding we return to in the Section General Discussion.

These results confirmed our prediction that the individual words in the training lists were mutually discriminable and individually identifiable. Performance was not perfect, leaving open the possibility that multisensory training could be used to enhance performance beyond that obtained with unisensory training. At the same time, performance was high enough to potentially demonstrate a reliable impeder effect with multisensory training.

There was also evidence that learning generalized to consonant identification in pre- and post-training CVCVC stimuli. This evidence was obtained both in terms of phonemes correct and in terms of phoneme equivalence classes correct. The consonants correct result strikes us as fairly remarkable, given that throughout training participants received no explicit feedback as to the phonemic strings they were learning and given the relatively brief training (generally fewer than three hours total). Also, perceptual learning frequently does not generalize or transfer to unlearned stimuli or to learned stimuli in different contexts (Nahum et al., 2010). The finding that paired-associates training generalized to consonant identification suggests that the level of perceptual learning induced by the paired-associates training task was not just at the level of whole nonsense words but reached to the level of phonemic categories or phonetic features, similar to results obtained previously using the same paradigm but for auditory training (Bernstein et al., 2013, 2014).

## Experiment 2: lipreading training with VA vs. VO stimuli

Having established performance levels in the VO Experiment 1, we next examined whether multisensory training with an acoustic speech stimulus would promote or impede VO perceptual learning. The literature reports examples of auditory stimuli promoting visual perceptual learning with non-speech stimuli (Shams and Seitz, 2008; Shams et al., 2011; Zilber et al., 2014). But our recent study of prelingually deaf adults with late-acquired cochlear implants showed that visual speech impeded auditory perceptual learning (Bernstein et al., 2014), while the same AV training did not impede and even promoted to some extent the auditory perceptual learning of adults with normal hearing. We hypothesized that a main factor between the groups was that the deaf adults had relied on vision for speech perception throughout their lives. When visual stimuli were available to them, they may have relied on their lipreading ability rather than using the concordance between visual and auditory speech to learn the patterns in the auditory stimuli. In addition, they may have relied on their ability to integrate their relatively poor auditory representations with visual speech representations to achieve better multisensory perception (Giraud et al., 2001; Moody-Antonio et al., 2005; Rouger et al., 2008; Huyse et al., 2013). Either perceptual strategy would be expected to reduce auditory perceptual learning.

In Experiment 2, we sought evidence for a similar effect but with normal-hearing adults. Here, our hypothesis was that multisensory training can impede unisensory perceptual learning when the target of training—in this case lipreading—is less proficient or a minor pathway for speech perception compared with the participant’s major perceptual modality—listening—with stimuli presented synchronously. Showing this to be the case in neurotypical adults would suggest that the impeder effect in the cochlear implant users was not due to their atypical perceptual experience but to a general propensity to rely on a primary source of stimulus information when it is available, even if the stimulus is highly degraded.

**Figure 5:**
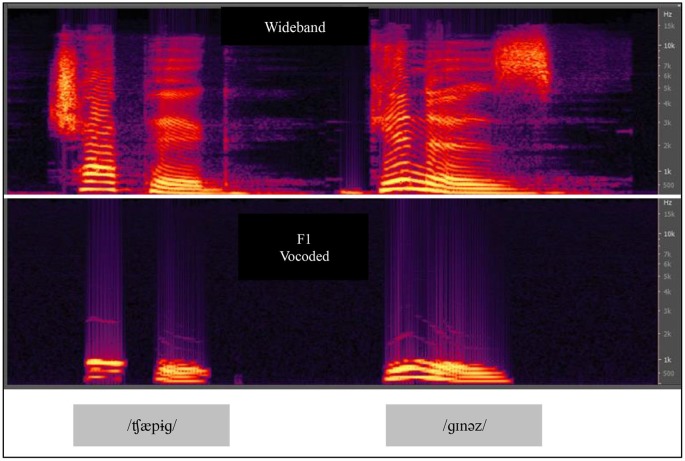
**Spectrograms of CVCVC speech stimuli, broadband and F1 vocoded**. The vocoded speech was used in Experiments 2 and 3. Two CVCVC stimuli (/ʧæpɨɡ/left, and /ɡɪnəz/right) are shown, the top row based on the recorded broadband speech, and the bottom output by the F1 vocoder.

In Experiment 2, we compared VO results from Experiment 1 with results obtained using VA stimuli for paired-associates training. We used an acoustic vocoder that we studied previously, labeled the F1 vocoder (Iverson et al., 1998). Figure 5 shows spectrograms of natural and F1 vocoded speech. The vocoder transformed broadband acoustic speech signals into 11 sinusoids each at the center frequency of a sixth-order band pass filter. The filters were spaced 75 Hz apart from 75 to 900 Hz and therefore approximately covered the range of the first speech formant. The energy passed by each band modulated a fixed frequency sinusoid at the center frequency of the pass band. The bands were equalized so that the natural amplitude tilt was removed. It was therefore a highly reduced acoustic speech signal. When the stimuli were presented for AO identification of the 22 initial English consonants in CV position and the 15 vowels in /h/-V-/d/ position, percent correct was 47.8% for consonants and 51.3% for vowels. When the video recordings of the same stimuli were presented VO, the results were 28.9% correct for consonants and 67.9% for vowels. Models of the lexicon were computed with these results, and the F1 vocoder was predicted to be less informative than lipreading for identifying words in a lexicon of approximately 31,000 words.

Experiment 2 entertained two possible outcomes: (1) vocoded speech could promote VO perceptual learning, because the speech was concordant and could assist trainees in discerning visual information in analogy with audiovisual training to promote auditory learning of vocoded speech; or (2) vocoded speech could impede VO perceptual learning, because even highly degraded acoustic speech may be used by itself or integrated with visual speech with scant learning of the visual speech *per se*, the actual target of the training.

### Methods

#### Subjects

The groups in this experiment were the VO trainees from Experiment 1, and the VA participants described earlier in the overall methods section.

#### Procedures

The procedures followed those described above, except that the paired-associates paradigm was carried out with the VA stimuli during training.

### Results

#### Lipreading screening scores

Independent samples *t-test* showed that the VA and VO groups did not differ in lipreading screening scores, *t*_(31)_ = 0.070, *p* = 0.945. Visual-only participants’ mean lipreading screening score was 13.4% correct, and VA participants’ mean lipreading screening score was 13.7%. Screening scores did not correlate with paired-associates training or test scores, but they did correlate with consonants correct in pre- (range across consonant initial, medial, and final positions, *r* = 0.170, *p* = 0.343 to *r* = 0.710, *p* = 0.000) and post-training CVCVC stimuli (range across positions from *r* = 0.388, *p* = 0.026 to *r* = 0.672, *p* = 0.000) and with lipreading tests at pre- (*r* = 0.871, *p* = 0.000) and post-training (*r* = 0.926, *p* = 0.000) times.

#### Paired-associates training scores

Figure 3 shows the time series of training and test scores for the VA and VO groups. The time series suggests that training performance improved across times for both groups, but VO training was more successful during training and test as the training progressed across time.

The final training block score of the four training lists was submitted to analysis with condition (VA, VO) as the between groups factor. Condition was not a reliable effect (*p* = 0.498). List was not reliable (*p* = 0.143), but their interactions was *F*_(3,29)_ = 4.349, *p* = 0.012, $\eta_{p}^{2}$ = 0.310. However, this interaction was not reliable for any of the individual contrasts across conditions. It does however support the impression of Figure 3 that there was improvement across time but its trajectory was different across VA vs. VO groups.

#### Test scores

Test scores were submitted to analysis with the four lists and two experimental groups (VO, VA) as the between groups factor. Condition was the only reliable factor, *F*_(1,31)_ = 10.177, *p* = 0.003, $\eta_{p}^{2}$ = 0.247. VO-trained participants were more accurate (mean = 82.3% correct) than VA-trained participants (mean = 64.9% correct). This result supports the hypothesis that—for normal-hearing adults—acoustic stimuli function as impeders while learning to lipread.

An additional analysis was carried out on the VA participants’ test scores. There was a reliable list effect, *F*_(3,10)_ = 5.015, *p* = 0.022, $\eta_{p}^{2}$ = 0.601. But within-subjects contrasts showed that the list effect is due to a drop in scores from List 3 to List 4, *F*_(1,12)_ = 8.929, *p* = 0.011, $\eta_{p}^{2}$ = 0.427. That is, there was no evidence that List 2 was learned better than List 1, or that List 3 was learned better than List 2, but there was a reliable drop from List 3 to List 4. Thus, although these participants were able to achieve on average test scores of 64.9%, they did not demonstrate any learning across lists in the same paradigm that reliably demonstrated learning in VO-trained participants.

#### Paired-associates training vs. test scores

The scores on the final block of training for each paired-associates list were subtracted from the test score for that list. These difference scores are good estimates of the relationship between learning in the training condition and subsequent test performance within participants. These scores were submitted to analysis with the four lists and two experimental groups (VO, VA) as the between groups factor. Condition was the only reliable factor, *F*_(1,31)_ =16.374, *p* = 0.001, $\eta_{p}^{2}$ = 0.346. VO-trained participants’ test scores were maintained between training and test (mean difference = 0 percentage points) while VA-trained participants’ scores dropped substantially (mean difference = −11.7 percentage points).

#### CVCVC consonant identification scores

Because there were correlations between lipreading screening scores and pre-training consonant identification scores, the lipreading screening scores were used as covariate in the analyses of CVCVC consonant identification during pre- and post-training tests.

First, scoring in terms of proportion consonants correct (see Table 2), pre- and post-training scores were submitted to analysis with the within-subjects factors of position (initial, medial, final) and test time (pre-, post-training) and between-subjects condition (VO, VA). Consonant position was a reliable main effect *F*_(2,29)_ = 97.097, *p* = 0.000, $\eta_{p}^{2}$ = 0.870. The lipreading screening score covariate was reliable, *F*_(1,30)_ = 30.912, *p* = 0.000, $\eta_{p}^{2}$ = 0.507, as was its interaction with consonant position, *F*_(2,29)_ = 8.685, *p* = 0.001, $\eta_{p}^{2}$ = 0.375. In pairwise comparisons, each position was significantly different from the other (*p* = 0.005) (initial = 32.0%, medial = 29.2%, final 19.6% correct). However, this scoring suggested that training had no effect on post-training scores.

Analyses for phoneme equivalence class scoring (see Table 2) used the same design. Again lipreading screening scores were used as a covariate, which was reliable, *F*_(1,30)_ = 20.544, *p* = 0.000, $\eta_{p}^{2}$ = 0.406. But using this scoring, there were three reliable main effects. One was position *F*_(2,29)_ = 35.828, *p* = 0.000, $\eta_{p}^{2}$ = 0.712, one was time of test, *F*_(1,30)_ = 17.016, *p* = 0.000, $\eta_{p}^{2}$ = 0.362, and one was condition, *F*_(1,30)_ = 6.169, *p* = 0.019, $\eta_{p}^{2}$ = 0.171. Because condition did not interact with time of testing, this analysis suggests that training, regardless of type (VO, VA) was a benefit to post-training consonant identification.

### Discussion

In Experiment 2, participants were trained in the paired-associates training task in VA or VO conditions. Training scores were not different across groups, but the VO groups’ VO test scores were higher than those of the VA group. Visual-auditory participants were significantly impeded in learning the VO stimuli. On average, their scores dropped 11.7 percentage points between training and test. Both groups improved their CVCVC consonant identification scores, although the improvement was reliable only with the phoneme equivalence class scoring.

These results support the hypothesis that the auditory speech impeded rather than promoted visual speech perceptual learning in the paired-associates task. The vocoded acoustic speech was designed to be highly degraded, but the visual speech was designed to be highly distinct and identifiable, which was confirmed in Experiment 1. Nevertheless, the possibility remained that the acoustic speech was actually much more informative than was suggested by the CV phoneme identification scores cited earlier (Iverson et al., 1998). In fact, we previously suggested based on computational modeling that multisyllabic stimuli could be quite well identified via the F1 vocoder (Iverson et al., 1998). Experiment 3 was carried out to determine how well the auditory only stimuli could be learned. If in fact they were easily learned and identified, the parallel to the previous impeder effect with cochlear implant patients (Bernstein et al., 2014) would be incorrect.

The finding that both VA and VO training resulted in pre- to post-training consonant identification improvements, even though the VA training impeded paired-associates learning is discussed in more detail in the Section General Discussion.

## Experiment 3: AO training

A group of participants was recruited to train with AO stimuli. They carried out the paired-associates training and test paradigm with AO stimuli but were tested with pre- and post-training tests using VO stimuli. An additional consideration for Experiment 3 was whether lipreading ability was associated with the different outcomes of paired-associates training with AO vs. VO stimuli. Therefore, for this experiment a subset of Experiment-1 VO trainees was matched in terms of individual lipreading screening scores to the participants who received AO training. Rather than statistically controlling for lipreading ability, this approach was used so that actual abilities were equated. As pointed out earlier, we doubt that the lipreading screening score continuum is merely a quantitative linear one but comprises qualitative differences, which likely correspond to important processing differences among lipreaders (Bernstein and Liebenthal, submitted).

### Methods

#### Subjects

The eight participants recruited for AO training were compared with eight Experiment-1 VO trainees matched on lipreading screening scores.

#### Stimuli

The stimuli during paired-associates training and testing were the F1 vocoded acoustic stimuli used in Experiment 2.

#### Procedure

The same protocol was used as in Experiments 1–2, except that paired-associates training and testing was AO.

### Results

#### Lipreading screening scores

The AO and VO means scores were compared and were no different according to an independent samples test, *t*_(14)_ = 0.589, *p* = 0.566. Both groups comprised poor lipreaders (AO lipreading screening mean score = 5.6%; VO lipreading screening mean score = 6.9%).

#### Paired-associates training scores

**Figure 6:**
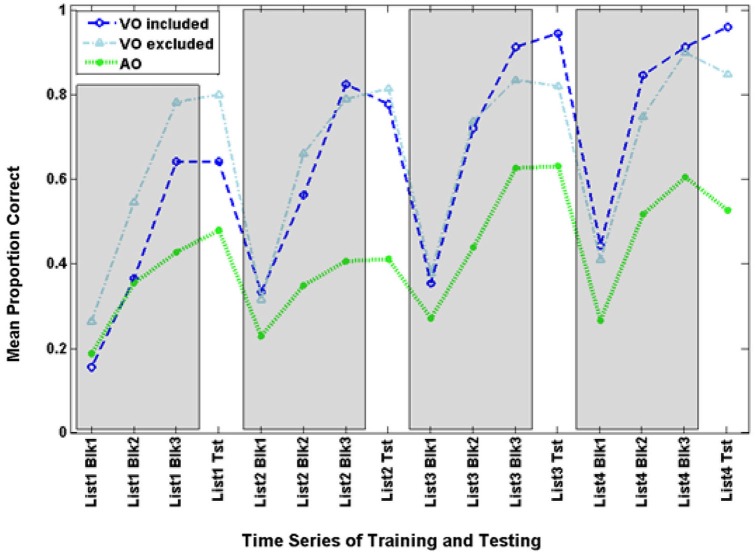
**Time series for the comparison VO and AO groups in Experiment 3 and the excluded VO participants**. Inclusion was based on lipreading screening scores that were matched with scores of AO participants.

Figure 3 shows the time series for AO participants and the full set of VO participants. Figure 6 shows the AO participants and the subset of VO participants who were compared with them in the analyses here (As requested by a reviewer, the figure also shows the excluded VO participants). The final training block score of the four training lists was submitted to analysis with condition (AO, VO) as the between groups factor. Condition was a reliable effect, *F*_(1,14)_ = 12.454, *p* = 0.003, $\eta_{p}^{2}$ = 0.471 (VO mean = 82.2% correct; AO mean = 51.6% correct). List was reliable, *F*_(3,12)_ = 10.179, *p* = 0.001, $\eta_{p}^{2}$ = 0.718, but the interaction of list by condition was not.

#### Paired-associates test scores

Test scores were submitted to analysis with list as the repeated factor and group as the between factor. In this case, group was the same as modality (i.e., AO, VO). Both were reliable, but their interaction was not. Visual-only participants’ test scores were higher than AO participants’, *F*_(1,14)_ = 9.798, *p* = 0.007, $\eta_{p}^{2}$ = 0.412 (VO mean = 82.9% correct; AO mean = 51.2% correct). The main list effect was *F*_(3,12)_ = 11.612, *p* = 0.001, $\eta_{p}^{2}$ = 0.744. The list effect was mainly attributable to improvement from List 2 to 3 (*p* = 0.000).

#### Training vs. test scores

The scores on the final block of training for each paired-associates list were subtracted from the test score for that list as estimates of the relationship between learning in the training condition and subsequent test performance within participants. These scores were submitted to analysis with the four lists and two experimental groups (AO, VO) as the between groups factor. There were no reliable effects. Neither group changed scores when they went from training to test in the paired-associates part of the experiment.

#### Pre- and post-training CVCVC and lipreading scores

We defer presentation of the analyses of these results to Experiment 5, in which we compare across all of the groups in this study. The AO group is considered there as a control group for the training task.

### Discussion

Experiment 3 examined whether the impeder effect obtained with vocoded acoustic speech in Experiment 3 could be attributed to vocoded acoustic speech affording more information than the visual speech. A group of participants was recruited for AO training, and they were matched on lipreading scores with members of the VO group from Experiment 1. Both groups comprised poor lipreaders. The results of the experiment clearly showed that even for poor lipreaders, the visual speech stimuli were the more informative: the AO group, which was trained and tested with AO stimuli, performed at significantly lower levels on training and testing across the four stimulus lists.

The VO participants’ results show that the paired-associates task can be learned even by poor lipreaders. The lipreading screening scores for the VO participants selected for comparison here ranged from 2 to 12%. Surprisingly, their mean training scores were 91% correct. We return to this point in the Section General Discussion.

The results in Experiment 3 support the hypothesis that stimuli presented via a trainees’ primary speech modality can impede learning by a lower rank modality. However, an alternative possibility is that the impeder effect is brought about by highly novel stimuli and not by relative perceptual rank. Experiment 4 was designed to test these alternatives using vibrotactile speech stimuli.

## Experiment 4: lipreading training with vibrotactile stimuli

A possible explanation for the impeder effect of vocoded acoustic speech in Experiment 2 is that vocoded speech is perceptually novel. Typically, experience is required in order to achieve more accurate perception with this type of stimulus (Davis et al., 2005; Hervais-Adelman et al., 2011; Wayne and Johnsrude, 2012; Bernstein et al., 2013). The novelty of the stimuli could be the cause of the impeder effect, not their modality.

We investigated this possibility using a vibrotactile vocoder. In our introduction above, we noted that a number of efficacy studies on the use of vibrotactile speech stimuli demonstrated improved lipreading following VT training. The vibrotactile vocoder used here (previously labeled *GULin*) (Bernstein et al., 1991) was implemented with a front-end acoustic vocoder with outputs of each vocoder channel as the driver signal for individual vibrators. Vibration characteristics were selected to match receptor characteristics for the volar forearm site of stimulation.

Vibrotactile stimuli are at least as, if not more, novel than vocoded acoustic speech. If vibrotactile stimuli do not impede or even promoted VO perceptual learning, this would support the view that the impeder effect is indeed strongly related to the perceptual ranking of the target training modality. In this case, lipreading would be expected to be more highly developed than vibrotactile speech perception, even if the trainee’s lipreading ability were measured to be zero for lipreading words in sentences. Even very poor lipreaders can benefit from visual speech (Ross et al., 2007), suggesting that sub-lexical phonemic or phonetic features contribute to audiovisual benefit and supporting the likelihood that vision has a higher rank than somatosensation for speech perception in normal-hearing adults.

### Methods

The approach in Experiment 4 was the same as that for the VA participants in Experiment 2, but VT stimuli were used during paired-associates training.

#### Subjects

Participants were recruited to be trained with VT stimuli. The VO participants from Experiment 1 were used to compare with the VT trainees.

#### Tactile stimuli

The vibrotactile vocoder (*GULin*) used here to present speech was previously described in an experiment that showed that with extended training it could be used *in combination with visual speech* to improve speech perception (Bernstein et al., 1991). Its filters were centered at 260, 392, 525, 660, 791, 925, 1060, 1225, 1390, 1590, 1820, 2080, 2380, 2720, and 3115 Hz, with respective bandwidths of 115, 130, 130, 130, 130, 130, 145, 165,190, 220, 250, 290, 330, 375, and 435 Hz. An additional highpass filter with cutoff 3565 Hz was also used. The signal energy from each filter was used to modulate the amplitude of a 250 Hz sinusoid signal driving an individual tactile stimulator.

The tactile array was configured as a 2 × 8 channel device with subminiature loudspeakers (PUI Audio Incorporated, Model AS01808MR-R) as the stimulators. Each was embedded in a foam mat, with spacing between loudspeakers of about 35 mm, and with stiff tactors (extending bars) epoxied to the diaphragms of each speaker. The signals were routed such that the highest-frequency channels were closest to the wrist. The stimulator mat was loosely wrapped to the left volar forearm with gauze and secured with a tubular elastic mesh. Participants were free to adjust the array position so that it felt comfortable to them.

In order to defeat the possibility of hearing acoustic radiation generated by the vibrators, participants wore circumaural headphones that presented 65-dB SPL pink noise.

#### Analyses

The analysis of Experiment 4 was carried out in two phases. First, the data from all participants were analyzed. However, additional analyses suggested the possibility that if a performance criterion were imposed such that performance was required to be moderately successful during paired-associates training, then analyses would show different results. Similar to our approach in the past (Bernstein et al., 2013, 2014), we imposed a 70.9% correct criterion on final training blocks, in this case for Lists 2–4. Participants who failed to achieve this criterion were screened out of the additional analyses.

### Results

#### Lipreading screening scores: full set

An independent samples *t-test* showed that the VT and VO groups did not differ in lipreading screening scores, *t*_(39)_ = 0.999, *p* = 0.324. Visual-only participants’ mean lipreading screening score was 13.4% correct, and VT participants’ mean score was 17.4%.

#### Paired-associates training scores: full set

Figure 3 shows the time series for the VT participants. The final training block score of the four training lists was submitted to analysis with condition (VT, VO) as the between groups factor. List was the only reliable effect, *F*_(3,37)_ = 13.633, *p* = 0.000, $\eta_{p}^{2}$ = 0.525. Scores improved across training. In simple contrasts, List 2 scores (mean = 76.6%) were higher than List 1 scores (mean = 66.2%), *F*_(1,39)_ = 13.579, *p* = 0.001, $\eta_{p}^{2}$ = 0.258; and List 3 scores (mean = 81.0%) were higher than List 2 scores, *F*_(1,39)_ = 4.518, *p* = 0.040, $\eta_{p}^{2}$ = 0.104; but List 4 scores (mean = 86.2%) were only marginally higher than List 3 scores *F*_(1,39)_ = 3.472, *p* = 0.070, $\eta_{p}^{2}$ = 0.082.

#### Paired-associates test scores: full set

Test scores were submitted to analysis with the four lists and two experimental groups (VT, VO) as the between groups factor. List was the only reliable factor, *F*_(3,37)_ = 5.777, *p* = 0.002, $\eta_{p}^{2}$ = 0.319, and the only list effect that was reliable in simple contrasts was an increase in scores from List 1 to List 2, *F*_(1,39)_ = 8.184, *p* = 0.007, $\eta_{p}^{2}$ = 0.173. The difference between groups, with mean VO scores of 82.3% correct and mean VT of 75.6% correct was not reliable, suggesting that the vibrotactile stimuli neither promoted nor impeded visual speech perceptual learning.

#### Paired-associates training vs. test scores: full set

The scores on the final block of training for each paired-associates list were subtracted from the test score for that list. These scores were submitted to analysis with the four lists and two experimental groups (VO, *n* = 20; VT, *n* = 21) as the between groups factor. In this analysis, group was not reliable, *p* = 0.088, and no other factors were reliable.

#### Pre- and post-training CVCVC scores: full set

The CVCVC identification scores were submitted to a repeated measures analysis with the repeated factors position (initial, medial, final) and test (pre, post), and the group factor (VO, VT). Lipreading screening scores were used as a covariate.

Scoring for consonants correct returned a reliable effect of the lipreading screening covariate, *F*_(1,38)_ = 30.454, *p* = 0.000, $\eta_{p}^{2}$ = 0.445. This was not surprising, as there was a range of individual lipreading scores. The only interaction with screening scores was with consonant position, *F*_(2,37)_ = 5.406, *p* = 0.009, $\eta_{p}^{2}$ = 0.226. As lipreading screening did not interact with condition, the interaction was not investigated further. Position was the only other reliable effect, *F*_(2,37)_ = 100.575, *p* = 0.000, $\eta_{p}^{2}$ = 0.845, and we defer further discussion of position to Experiment 5.

Scoring for equivalence classes correct returned a reliable effect of the lipreading screening covariate, *F*_(1,38)_ = 14.671, *p* = 0.000, $\eta_{p}^{2}$ = 0.279. The only interaction with screening scores was a three-way with position and test, *F*_(2,37)_ = 6.623, *p* = 0.003, $\eta_{p}^{2}$ = 0.264. As lipreading screening did not interact with condition, the three-way interaction was not investigated further. Position, *F*_(2,37)_ = 68.881, *p* = 0.000, $\eta_{p}^{2}$ = 0.788, test, *F*_(1,38)_ = 17.390, *p* = 0.000, $\eta_{p}^{2}$ = 0.314, and condition *F*_(1,38)_ = 6.798, *p* = 0.013, $\eta_{p}^{2}$ = 0.152 were reliable. Position effects were similar to those in Experiments 1–3, and we defer further discussion of them to Experiment 5. Scores increased from pre- to post-training tests, and the VO group was more accurate overall. Because there was not an interaction between test time and group, we do not consider the test time main effect further.

#### Subset of results from participants able to learn to criterion

**Figure 7:**
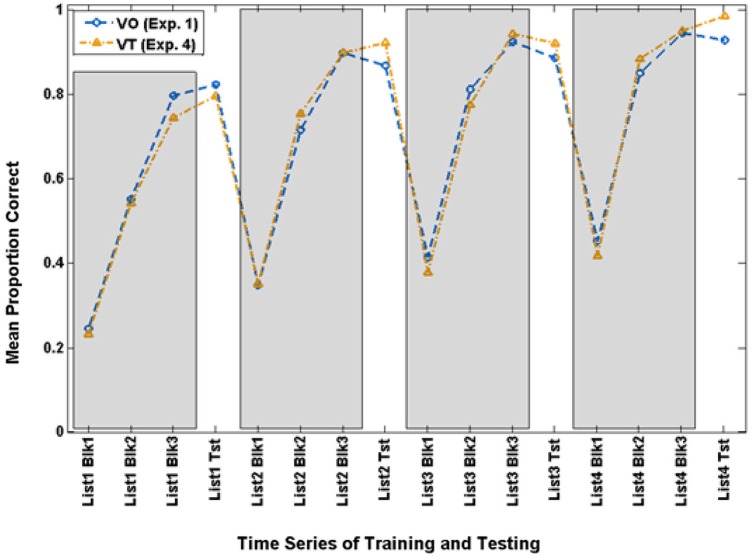
**Time series of paired-associates mean scores of VO and VT participants who achieved criterion (70.9% correct) on Block 3 of Lists 2–4 and were used for the extended analyses in Experiment 4**.

Careful examination of the training scores for the VO and VT participants suggested that the overall results reported just above might be obscuring a vibrotactile promoter effect. In the past, we have analyzed results across groups after setting a criterion for success during training. Therefore, we set a criterion level of 70.9% during the final training block of Lists 2–4 as the lowest level of training performance that qualified as successful training. This resulted in retaining 14 of the 20 VO participants and 12 of the 21 VT participants. Figure 7 shows the time series for training and testing with paired-associates for these subgroups.

These two groups did not differ in terms of lipreading screening scores, *t*_(24)_ = 1.449, *p* = 0.160. There was however a strong correlation between screening scores and the List 1 VO test scores, *r*_(26)_ = 0.656, *p* = 0.000, but not with other lists. Lipreading screening scores were used as covariates in these analyses.

The analysis of the paired-associates training scores showed that the only reliable effect was list, *F*_(3,21)_ = 11.361, *p* = 0.000, $\eta_{p}^{2}$ = 0.619, which was also reliable for its linear (*p* = 0.000) and quadratic (*p* = 0.014) contrasts.

The analysis of the paired-associates test scores showed that there was a main effect of the lipreading screening score covariate, *F*_(1,23)_ = 4.532, *p* = 0.044, $\eta_{p}^{2}$ = 0.165. The main effect of condition was not reliable (*p* = 0.457). List was *F*_(3,21)_ = 15.679, *p* = 0.000, $\eta_{p}^{2}$ = 0.691, and it was reliable for a linear trend, *p* = 0.000. List also interacted with lipreading screening score, *F*_(3,21)_ = 7.683, *p* = 0.001, $\eta_{p}^{2}$ = 0.523.

**Figure 8:**
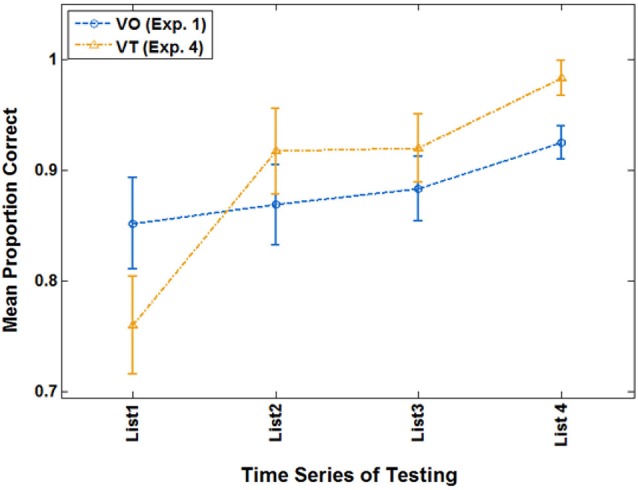
**Cross-over interaction for VO test scores in the extended analysis of Experiment 4 with participants who were at criterion or better during paired-associates training**.

Of primary interest here, there was a cross-over interaction between list and condition, *F*_(3,21)_ = 3.214, *p* = 0.044, $\eta_{p}^{2}$ = 0.315. Simple contrast tests for trend showed that condition interacted only with a linear trend, *F*_(1,23)_ = 4.560, *p* = 0.044, $\eta_{p}^{2}$ = 0.165. As Figure 8 shows, both groups improved across lists, but the VT group did so more steeply. On their final VO test, VT group mean test scores were 98.2% correct, and mean VO group test scores were 92.5%.

There were no reliable effects in the analysis of the training vs. test scores. Nor were there any condition effects that involved the pre- and post-training consonant identification scores or lipreading (see Table 2).

### Discussion

Experiment 4 showed that a novel vibrotactile stimulus delivered while training was carried out on paired associations did not impede learning as demonstrated by omnibus analyses. Furthermore, when participants were selected from each group based on the criteria that their last training block on Lists 2–4 was at 70.9% or greater (retaining in 12 VT and 14 VO participants) the results showed that there was an effect of the vibrotactile stimuli: VT participants learned faster than VO participants. Thus, holding criterion constant across VT and VO groups, the vibrotactile stimuli promoted visual speech perceptual learning in the paired-associated paradigm. These results are discussed in more detail in the Section General Discussion.

## Experiment 5: no-training control

Although there was evidence suggestive of generalization from the paired-associated training paradigm to consonant identification on the post-training task with untrained CVCVC stimuli, this could not be assured without testing a group of participants who received no paired-associates training experience. Therefore, no-training control participants were tested only on the pre- and post-training consonant identification and lipreading tests. Their results were compared to those from the previous four experiments.

### Methods

#### Participants

Thirteen no-training control subjects were recruited and compared here with those of all the previous participants.

#### Procedure

The participants were tested on two separate days on the CVCVC consonant identification and sentence lipreading tasks. The procedures for these tests were the same ones used for pre- and post-testing in Experiments 1–4.

#### Analyses

The approach was to first compare the two groups *without* visual training experience (i.e., controls and AO participants). When they were shown to be similar, their results were pooled. Then the groups *with* visual training experience (VO, VA, and VT) were compared, and subsequently their results were pooled. Then the groups with and without visual training experience were compared.

### Results

#### Pre- and post-training CVCVC scores

Table 2 gives the CVCVC scores in terms of proportion consonants correct and in terms of proportion phoneme equivalence classes correct for all of the groupings analyzed in this experiment.

The first analysis addressed whether there was any difference between the no-training controls and the AO participants from Experiment 3 in an analysis with within factors consonant position (initial, medial, final) and test time (pre-, post-), with lipreading screening as a covariate. There were no main effects or interactions involving group. The next analysis addressed whether there were differences among the three groups with visual training (Exp. 1, VO; Exp. 2, VA; and Exp. 4, VT). The consonants correct scoring returned no reliable effects. The analysis based on phoneme equivalence classes returned a main effect of condition, *F*_(2,50)_ = 4.259, *p* = 0.020, $\eta_{p}^{2}$ = 0.146, but condition did not interact with time of testing. There was therefore motivation to combine results according to whether participants had visual training experience (VA, VT, and VO) or did not (AO, no-training controls).

Using consonants correct scoring, the two groups were evaluated with lipreading screening scores as the covariate, and the within factors time of test (pre-, post), and consonant position (initial, medial, final). No reliable effects were returned involving the group factor. Position, *F*_(2,71)_ = 236.636, *p* = 0.000, $\eta_{p}^{2}$ = 0.870, lipreading screening, *F*_(1,72)_ = 57.273, *p* = 0.000, $\eta_{p}^{2}$ = 0.942, and their interaction, *F*_(2,72)_ = 15.806, *p* = 0.000, $\eta_{p}^{2}$ = 0.308, were reliable.

**Figure 9:**
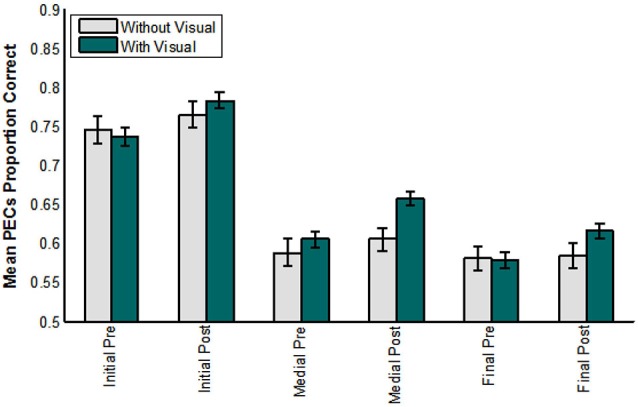
**Pre- vs. post-training consonant identification scored in terms of phoneme equivalence classes (PECs)**. The participants who did not receive visual training (−V) comprised those in the AO and control groups. The participants who did receive visual training (+V) comprised the VO, VA, and VT groups. (Initial = initial consonant; Medial = medial consonant; Final = final consonant).

Figure 9 shows the results across groups (with, without visual training experience) over test time (pre-, post-), and for each consonant position (initial, medial, final) scored in terms of phoneme equivalence class scoring. The analysis of these factors showed that position was reliable, *F*_(1,71)_ = 145.471, *p* = 0.000, $\eta_{p}^{2}$ = 0.804. Test time was reliable, *F*_(1,72)_ = 26.779, *p* = 0.000, $\eta_{p}^{2}$ = 0.271, as was its interaction with lipreading screening *F*_(1,72)_ = 5.164, *p* = 0.026, $\eta_{p}^{2}$ = 0.067.

Most importantly, test time interacted with the group factor, *F*_(1,72)_ = 5.922, *p* = 0.017, $\eta_{p}^{2}$ = 0.076. Participants without visual training experience had mean phoneme equivalence scores of 76.2% correct at pre-testing and 78.3% correct at post-testing. Participants with visual training experience had mean phoneme equivalence class scores of 79.4% at pre-testing and 84.8% at post-testing (not adjusted for the covariate).

#### Pre- and post-training lipreading scores

Pre- and post-training lipreading scores were approached through the same two analysis steps as those for the CVCVC phoneme identification scores. There were no reliable effects related to time of testing.

### Discussion

The no-training controls and the AO-trained participants were found to be similar in their pre- and post-training consonant identification test scores, and the participants with any type of visual training experience were found to be similar to each other. The pooled groups (with, without visual training) were significantly different in that those with visual training significantly improved on consonant identification between pre- and post-training test periods. This result implies that the generalization from paired-associates training was not attributable to task learning: had it been, we would have expected a pre- to post-training gain with AO training. Instead, some aspects of experience with visual speech during training were required to achieve generalization. This result sends a cautionary message: if generalization occurs following multisensory training with an impeder or a promoter stimulus or following unisensory training, the effect seems most straightforwardly attributable to aspects of the visual experience that were not explicitly manipulated in this study.

## General discussion

The study reported here shows that multisensory training is not necessarily advantageous for unisensory perceptual learning, although it can be. A speech stimulus in one modality that accompanies the speech training that targets another modality can be an impeder or a promoter of unisensory perceptual learning, depending on its rank. We use the term “rank” to refer to the relative capability or development of a sensory pathway for speech perception within an individual. Normal-hearing individuals rely on auditory speech perception and have to varying extent ability to perceive visual speech. They are not expected to have experienced vibrotactile vocoded speech, but they have experienced somatomotor feedback from their own speech production, including stimulation from laryngeal vibration, the breath stream, and kinesthesia. Thus, vibrotactile speech stimuli are of tertiary rank.

In this study, visual speech perception was the training target. In Experiment 1, VO training on paired associations between CVCVC spoken nonsense words and nonsense pictures was used to show that the visual speech stimuli can be learned even by poor lipreaders, and that training carried over to identification of the consonants in untrained CVCVC stimuli. Experiment 2 tested whether multisensory training would impede or promote unisensory learning. Vocoded acoustic speech stimuli were presented in synchrony with the visual speech during paired-associates training. But testing was with VO stimuli. In this experiment, VO paired-associates test scores were steeply lower than the VA training scores, implying that the vocoded speech impeded visual learning. However, there was also indication that the training did benefit identification of consonants in untrained CVCVC stimuli. Experiment 3 was designed to test whether the impeder effect of acoustic speech during paired-associates training in Experiment 2 was due to the acoustic speech affording more information than the visual speech. The vocoded acoustic speech from Experiment 2 was presented alone during the paired-associates paradigm. A subset of VO training results from Experiment 1 trainees matched on lipreading screening scores were used in comparison. Both groups comprised poor lipreaders. The results showed that performance on the AO stimuli was much lower than on the VO stimuli during the paired-associates paradigm. Experiment 4 tested the hypothesis that novelty, rather than the perceptual rank, drives the impeder effect. Vibrotactile vocoder stimuli were presented in synchrony with visual speech during the paired-associates training, followed by VO tests. In overall comparisons between VT and VO trainees there was no evidence that the vibrotactile stimuli impeded learning. When results were examined using only those participants whose training scores on the paired-associates passed a criterion of least 70.9%, there was evidence that the vibrotactile stimuli actually promoted VO learning. In Experiment 5, a no-training control group was tested on pre- and post-training consonant identification and also on lipreading sentences, which had also been tested in the previous experiments. Results showed that all types of visual experience (i.e., VO, VT, and VA) were of benefit to consonant identification in the post-training task, and scores were higher than those obtained by participants who did not receive visual training experience (AO, control). Lipreading of words in sentences was unaffected by any of the conditions in this study.

We discuss these results in terms of their implications for the use of multisensory stimuli to promote or impede perceptual learning. The term “perceptual learning” is used here to mean durable changes in perception that improve the ability to respond accurately to stimuli (Goldstone, 1998).^3^ We extend our discussion to the question of how a multisensory adaptation of RHT predicts the pattern of results obtained here and in our previous studies (Bernstein et al., 2013, 2014) using the paired-associates paradigm with auditory perceptual learning as the target.

### Promoter vs. impeder stimuli

We have demonstrated that, depending on the stimulus and the perceiver, multisensory training can impede or promote learning within a training paradigm. We propose two generalizations or principles to predict when a stimulus acts as a promoter or an impeder. These principles depend on the rank of the input processing sensory system in relationship to the perceptual task and the perceiver’s perceptual experience.

Principle 1: *Stimuli presented to the trainee’s primary perceptual pathway will impede learning by a lower-rank pathway*.

Principle 2: *Stimuli presented to the trainee’s lower rank perceptual pathway will promote learning by a higher-rank pathway*.

Principle 1 is demonstrated by Experiment 2, in which VA paired-associates training led to lower VO test scores, even though the VO stimuli afforded more information than the AO stimuli (see Figures 3, 5), and even though the participants were poor lipreaders. This principle was also demonstrated in Bernstein et al. (2014, Exp. 1), in which prelingually deaf adults whose higher rank system for speech perception is vision received paired-associates training with the goal of improving unisensory auditory perception. The cochlear implant users’ AO test scores were always steeply lower following AV training than following AO training. In both experiments, the impeder effect was strong even though the target stimuli were shown to be adequate for the participants to perform the unisensory perceptual task.

Principle 2 is the more commonly reported one in the literature (Weisenberger et al., 1989; Eberhardt et al., 1990; Bernstein et al., 1991, 2013, 2014; Waldstein and Boothroyd, 1995; Kishon-Rabin et al., 1996; Pilling and Thomas, 2011; Wayne and Johnsrude, 2012). In normal-hearing adults, visual speech stimuli have been shown to promote auditory perceptual learning, and in the same population, vibrotactile speech stimuli have been shown to promote visual speech perceptual learning. In Experiment 4, vibrotactile speech promoted visual perceptual learning among participants whose training scores were at or above criterion. There was also no evidence that the vibrotactile stimuli impeded learning when all the VO participants were compared with all the VT participants. Inarguably, vibrotactile stimuli are novel and of lower rank than visual speech stimuli to normal-hearing perceivers.

Principle 1 has the interesting implication that the failure to develop good lipreading on the part of most normal-hearing individuals could be attributable at least in part to auditory stimuli impeding learning. Expert lipreading by deaf individuals shows that visual stimuli present information or features that normal-hearing individuals typically do not learn (Bernstein et al., 2000, 2001; Mohammed et al., 2005; Auer and Bernstein, 2007). The finding that there were participants who scored close to zero on the lipreading screening test here but were able to learn the VO stimuli in paired-associates training to a high level of accuracy (Experiments 1 and 4) supports the conclusion that poor lipreading is not necessarily due to insensitivity to visual speech information. Given that visual speech stimuli are ubiquitous during face-to-face conversation, and given the general expectation that perceptual learning is responsive to the statistical properties of stimuli in the environment (Saffran et al., 1996; Abla and Okanoya, 2009; Shams and Kim, 2010), the failure to learn available visual features on the part of most normal-hearing individuals could be highly related to auditory stimuli acting as impeders against learning to lipread.

There are some possible counter-examples to Principle 2. For example, Huyse et al. (2013) showed that by reducing the clarity of visual speech in stimulus blocks comprising VO, AO, and audiovisual speech that speech perception was weighted more strongly towards the auditory stimuli in children with cochlear implants and ones with normal hearing. The weighting towards auditory speech is not surprising in normal-hearing children. The result with cochlear implant children is potentially counter to Principle 2. However, the deaf children received their implants by at least three years of age, and most had used their implant for quite a few years, so auditory perception would be expected to be of higher rank in those children than in the deaf adults we studied who had received their implants late. Nevertheless, the Huyse et al. (2013) study did show that perceptual weighting can be affected by adjusting visual clarity.

Another potential counter-example to Principle 2 comes from studies that demonstrate weighting of non-speech perception towards the modal component in whichever stimulus is relatively more informative (Ernst and Banks, 2002; Alais and Burr, 2004). For speech, this was demonstrated in a neuroimaging experiment with audiovisual stimuli. When noise vocoding was applied to acoustic speech, and filtering was applied to video speech, functional connectivity of the superior temporal sulcus was greater with the cortex that represented the more reliable auditory vs. visual stimuli (Nath and Beauchamp, 2011). However, this imaging study did not involve perceptual learning. Research on re-weighting is needed within the context of multisensory training. Functional connectivity adjustments in response to magnitude of modal information seems an optimal neural response, but one that actually may need to be overcome under circumstances such as a neural prosthesis that affords new yet-unlearned stimulus information (see below).

### A multisensory RHT account of promoter vs. impeder effects

The principles we proposed above are framed in terms of perceptual rank of the stimuli defined in terms of the perceiver’s past experience. Those principles invoke neural pathways, but they are concerned mostly with relationships among types of stimuli. We have previously discussed how RHT, an integrated behavioral and neural account of unisensory perceptual learning (Ahissar and Hochstein, 2004; Ahissar et al., 2009), can account for the case of auditory learning by normal-hearing adults and by prelingually deaf adults with late-acquired cochlear implants (Bernstein et al., 2013, 2014). We briefly review multisensory RHT and consider a mechanism that would support impeder vs. promoter stimulus effects.

The *hierarchy* in RHT refers to the cortical hierarchical organization of sensory-perceptual pathways (Felleman and Van Essen, 1991; Kaas and Hackett, 2000; Kral and Eggermont, 2007). Although perceptual pathways are not strictly hierarchical, their higher cortical levels typically show selectivity for increasingly complex stimuli as well as an increasing tolerance to stimulus transformation and increasing response to perceptual category differences (Hubel and Wiesel, 1962; Ungerleider and Haxby, 1994; Logothetis and Sheinberg, 1996; Binder et al., 2000; Zeki, 2005; Obleser et al., 2007). According to unisensory RHT, immediate perception relies on already-established higher-level representations in the bottom-up unisensory-perceptual pathway (Ahissar and Hochstein, 1997; Kral and Eggermont, 2007; Ahissar et al., 2009). When a *new* perceptual task needs to be carried out, naïve performance is initiated on the basis of immediately available high-level perception. However, if the task cannot be readily performed with the existing mapping of lower-level to higher-level representations, and/or if there is incentive to increase the efficiency of task performance, then perceptual learning may occur.

Perceptual learning at the neural level is by definition the access to and remapping of lower-level input representations to higher-level representations. The rapidity of perceptual learning suggests that the lower-level representations exist to be remapped. Notably, RHT posits that perceptual learning requires “perception with scrutiny.” In order to learn, a backward (top-down) search from a higher level of the representational hierarchy must be initiated to access lower-level representations where information is available to be mapped to higher-level representations. A more effective forward mapping can then be made in terms of altered convergence and/or divergence patterns within existing neural networks (Jiang et al., 2007; Kral and Eggermont, 2007; Ahissar et al., 2009).

In our approach to multisensory RHT (Bernstein et al., 2013, 2014), backward search can also take place from one perceptual system to another. So, perceptual distinctions that are available through one modality can guide scrutiny of the representations of another modality. This cross-modality backward search is possible because of a highly interconnected brain that affords cross-modal scrutiny. Indeed, the evidence is extensive for the sheer diversity and extent of cortical and subcortical multisensory connections (e.g., Foxe and Schroeder, 2005; Ghazanfar and Schroeder, 2006; Driver and Noesselt, 2008; Kayser et al., 2012). That is, neural resources are available for higher-level representations in one sensory system to gain access to lower-level representations in a different sensory-perceptual system, as well as for low-level cross-sensory connections to activate early areas (Ghazanfar et al., 2008; Falchier et al., 2012).

In addition, multisensory, amodal representations can gain access to lower-level unisensory ones (e.g., Calvert et al., 1999; Nath and Beauchamp, 2011). However, if amodal representations are more strongly associated with their primary or higher rank sensory input pathway, then reverse search would be predicted to be more strongly directed along the primary or higher rank pathway, even if that pathway is incorrect for the learning task.

The impeder effect would arise when backward search is initiated along the incorrect but more highly developed or connected pathway. Even though the trainee knows that the goal is to learn modal representations of a lower rank stimulus, its reverse pathway affords less developed representations to guide reverse search. Specifically, in Experiment 2 under VA training conditions with the goal of VO learning, under-developed reverse search along the normal-hearing trainees’ visual pathway would compete with multisensory integration and reverse search along the primary auditory pathway. Likewise, under AV training conditions with the goal of AO learning, under-developed reverse search along the cochlear-implant trainees’ auditory pathway would compete with multisensory integration and reverse search along the visual pathway.

This explanation is consistent with previous auditory RHT explanations (Kral and Eggermont, 2007; Kral and Sharma, 2012) for why cochlear implants are less effective in prelingually deafened individuals who receive them beyond the first 3 or 4 years of age (Wilson et al., 2011). According to Kral and Sharma (2012, p. 117), prelingual deafness is associated with abnormal connectivity, immaturity of auditory cortical areas, cross-modal recruitment of some auditory areas for non-auditory function, but “The presence of residual plasticity in late-implanted, prelingually, deaf subjects should in principle allow levels of speech performance comparable to early-implanted children after longer periods of experience with the implant. However, late implanted subjects continue to show poor speech recognition and auditory performance even after long durations of implant use.” These deficits are at least partially attributable to the absence of, or poorly developed, auditory speech representations to guide reverse search and remapping of the auditory features made available by the cochlear implant. Kral and Eggermont (2007, p. 263) suggest that, “As (supposedly) auditory categories cannot develop in deafness, the decreased plasticity in the auditory cortex cannot be compensated and directed by top–down modulatory influences. This developmental decrease in synaptic plasticity together with the absence of top–down mechanisms leads to a decrease in general ability to learn”.

Here, we are suggesting that a reverse search during training may be initiated through the sensory pathway with more highly developed representations rather than the one targeted by training goals. A reliance on the more highly developed pathway for reverse hierarchy search would result in visual stimuli impeding auditory perceptual learning in prelingually deaf adults with late-acquired implants and in auditory stimuli impeding visual perceptual learning in normal-hearing adults. On the other hand, reverse search through a higher rank system could be guided by a lower-rank system that presents modal features that are concurrent with or correlated with the to-be-learned stimulus features.

### Concurrent visual and acoustic or vibrotactile speech features

In order for our multisensory RHT to be correct, there must be concurrent multisensory stimuli that afford correlated information that can be used to discern and remap modal features. Audiovisual speech does afford correlated stimulus information (Jiang et al., 2002; Schroeder et al., 2008; Jiang and Bernstein, 2011; Schwartz and Savariaux, 2014) that is naturally available to perceivers. For example, easy visual distinctions such as “*p*” vs. “*t*”, which are difficult auditory distinctions for the cochlear implant user, are available to guide discernment of distinct auditory representations and thereby promote learning.

Because auditory stimuli impede visual learning, we used vibrotactile speech in Experiment 4. The vibrotactile stimuli were generated directly from acoustic vocoded speech and were therefore expected to present information correlated with visual speech. The obtained promoter effect supports the conclusion that the stimuli contained such useful information. In previous unpublished research we showed that the perceptual structure of acoustic vocoded speech can be used to predict successfully the perceptual structure of vibrotactile vocoded speech (Kello and Bernstein, 2000). Prosodic speech patterns are readily available via vibrotactile stimuli (Bernstein et al., 1989; Navarra et al., 2014) and could have contributed here to perceiving the fine structure of the CVCVC training stimuli.

### Perception biased by experience

In our previous study, cochlear implant users were most accurate for identifying initial consonants in CVCVCs, and normal-hearing adults were most accurate for medial consonant identification, before and after training (Bernstein et al., 2014, Table 2). Lipreaders are in general most accurate for initial consonants in CVCVC stimuli, and this is true whether they are deaf or hearing (Auer and Bernstein, in preparation). Apparently, the initial consonant affords the most information to the lipreader because co-articulatory gestures tend to obscure visibility of medial consonants. However, auditory perception can be more accurate for medial consonants, because in the VCV position, consonant information is distributed across the preceding and following vowel transitions (Stevens, 1998). In this study, Figure 4 (Table 2) shows that initial consonants were identified most accurately followed by medial and final consonants. Figure 9 shows that this pattern persisted across training, although there were gains made across positions. These results support further our previous conclusion about the visual bias that cochlear implant users bring to auditory speech perception. This bias seems to be related directly to the visibility of visual speech information independent of visual speech experience.

### Paired-associates training in relationship to pre- and post-training tests

By statistically adjusting for individual lipreading ability, analyses showed that trainees who received any type of visual training experience (i.e., VO, VA, or VT) improved their identification of consonants in untrained CVCVC stimuli in contrast with participants who did not experience any training with visual speech (i.e., no-training controls or AO).

The improvements in consonant identification are somewhat surprising in that at no time in the study did participants receive any feedback regarding the identity of the phonemes in the visual stimuli. The training protocol by itself using AO stimuli did not improve pre- and post-training consonant identification (Experiment 3). Therefore, some aspect of training with visual speech improved response accuracy, but what that was cannot be inferred from this study. The finding suggests that participants had sufficient implicit knowledge of visual speech phonemes to tune their responses based only on their experience in the training paradigm.

### Lipreading ability in relation to visual speech perceptual learning

**Figure 10:**
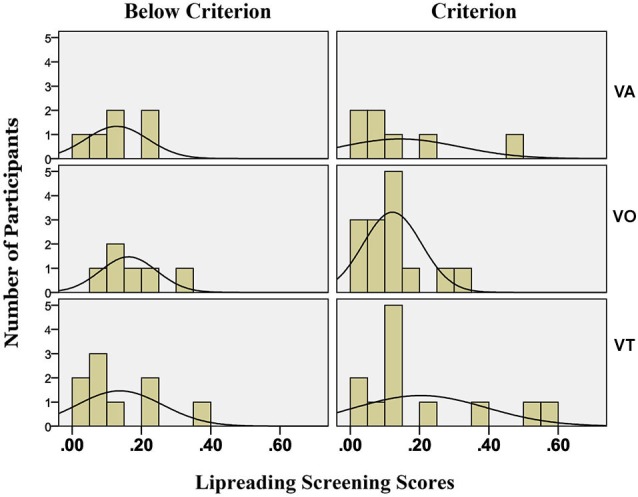
**Histogram of numbers of participants sorted on whether they achieved criterion during training (left column) or not (right column)**. Row 1, VA; Row 2, VO; Row 3, VT. Normal curves are fitted to each distribution.

One of the concerns in evaluating the results here was whether initial lipreading ability would control learning in the paired-associates paradigm. But results showed that initial lipreading ability was not a controlling factor for paired-associates learning, although it did correlate with the untrained consonant identification and lipreading task scores. Figure 10 shows histograms of the lipreading screening scores for the three groups that received training with visual stimuli (VA, VO, VT). The column labeled “below criterion” shows the participants who were unable to achieve the score of 70.9% correct on Block 3 of Lists 2–4. The “criterion” column shows those who achieved criterion or better. In both columns, there were participants with screening scores at or below approximately 20% words correct. Both columns show that the overall distributions of lipreading screening scores were approximately the same.

A question that the current study cannot answer is whether learning particular recordings of nonsense words carries over immediately to untrained recordings, and whether carryover is related to lipreading ability. Individual talkers do vary in visual intelligibility (Bernstein et al., 2000; Auer and Bernstein, in preparation), so training on one talker might not generalize to a range of talkers with varied intelligibility. Previous auditory speech training experiments examined generalization based on learning with one or a variety of talkers and showed talker-specific effects (Nygaard and Pisoni, 1998). We would expect talker-specific effects with visual speech training. In general, perceptual learning is expected to be more robust but require more training with multiple tokens of each stimulus (Nahum et al., 2008).

The current study failed to produce any evidence that lipreading sentences improved with paired-associates training. Lipreading sentences is a very stable ability (Bernstein et al., 2001). Nevertheless, improvements on the consonant identification task might imply that improvements should carry over to lipreading words. Phoneme scoring of the sentence responses could show that perception was more accurate even though whole word responses were not. However, expert lipreading might be organized somewhat differently than poor lipreading. One possibility is that good lipreading depends on whole-word visual speech representations (Bernstein and Liebenthal, submitted). A dual stream organization for more skilled lipreaders would be expected to have more automatized access to certain lexical items as well as need for phonological processing. A less skilled lipreader might be expected to have less access to automatized word processing and to rely typically on phonetic or phonemic category information. Training on a set of nonsense words would not be expected to improve perception of whole real words through an automatized pathway.

### Practical or clinical implications

There are many potential applications of multisensory training that could improve unisensory perceptual learning such as learning a new contrast in a foreign language, and learning to use a neural prostheses or a novel form of sensory substitution (Bradlow et al., 1999; Merabet et al., 2005; Hazan et al., 2006; Proulx et al., 2014). Clearly, understanding general principles of multisensory perceptual learning could speed progress in developing clinical or other practical applications.

Our proposed principles for identifying when a stimulus might be a promoter vs. an impeder imply that novel stimuli such as vibrotactile speech could be important in developing an effective toolkit for multisensory training. In particular, vibrotactile speech stimuli, being of lower rank, could be designed to guide trainees towards available but unlearned distinctions or features in auditory or visual speech. Such applications could make use of previous experiments on vibrotactile speech stimuli that were carried out at various times throughout the twentieth century with the goal to develop stimuli to augment speech perception in individuals with hearing loss (e.g., Gault, 1924; Kirman, 1973; Reed et al., 1982; Eberhardt et al., 1990; Bernstein et al., 1991; Weisenberger et al., 1991). This line of research was overtaken by development of cochlear implants, which were more effective for many deaf individuals, particularly young children (Miyamoto et al., 1995) and post-lingually deafened adults who experience high levels of benefit with their implant (Wilson et al., 2011). Indeed, current knowledge about vibrotactile speech perception could be used to bootstrap development of vibrotactile stimuli designed primarily for training regimes rather than as neural prostheses.

## Summary and conclusions

Multisensory training with speech stimuli can promote or impede unisensory perceptual learning. Two principles are proposed to account for multisensory stimulus effects: (1) *Stimuli presented to the trainee’s primary perceptual pathway will impede learning by a lower-rank pathway;* and (2) *Stimuli presented to the trainee’s lower rank perceptual pathway will promote learning by a higher-rank pathway*. Multisensory RHT suggests that the impeder vs. promoter effects may arise due to reverse search during training. A reverse search during training may be initiated through the sensory pathway with more highly developed representations rather than the one targeted by training goals. A reliance on the more highly developed pathway for reverse hierarchy search would result in visual stimuli impeding auditory perceptual learning in prelingually deaf adults with late-acquired implants and in auditory stimuli impeding visual perceptual learning in normal-hearing adults. On the other hand, reverse search through a higher rank system could be guided by a lower-rank system that presents modal features that are concurrent with or correlated with the to-be-learned stimulus features. If these suggestions are true, along with our proposed principles for multisensory effects during training, then they can be applied to achieve faster and more efficient training protocols in areas such as second language training, use of a neural prosthesis, and sensory substitution. Stimuli delivered to the somatosensory pathway, a pathway typically of lowest rank for speech, could be highly effective in multisensory speech training paradigms.

## Conflict of interest statement

The authors declare that the research was conducted in the absence of any commercial or financial relationships that could be construed as a potential conflict of interest.

## Footnotes

^1^We use the terms “visual-auditory” and “visual-tactile” to indicate that the first modality is the training target.

^2^http://wiki.cnbc.cmu.edu/Novel_Objects

^3^A distinction has been made between learning unfamiliar speech to improve understanding vs. tuning a phonetic category boundary along an acoustic continuum (Samuel and Kraljic, 2009). The results here are examples of the former, as is learning a contrast in a foreign language (Bradlow et al., 1999; Hazan et al., 2006). The phenomenon known as “recalibration” (Vroomen et al., 2007; Vroomen and Baart, 2012) is an example of the latter. Recalibration involves a short-lived change in speech perception that turns an ambiguous stimulus token into an unambiguous or less ambiguous example of a phonemic category. Perceptual learning of the type demonstrated with our paired-associates task carries over from list to list across days and also to untrained stimuli in a different task presented on a different day. In addition, the stimuli are not ambiguous in the sense that they afford the necessary information to carry out the tasks of paired-associates learning and consonant identification with untrained stimuli.
